# Determinants of adoption of quality protein maize varieties in Misrak Badewacho District, Southern Ethiopia: policy implications

**DOI:** 10.3389/fnut.2024.1467632

**Published:** 2025-01-06

**Authors:** Yilma Jambo, Alemu Daka, Berhanu Nega

**Affiliations:** ^1^Department of Rural Development and Agricultural Extension, College of Agriculture and Natural Resources, Madda Walabu University, Bale Robe, Ethiopia; ^2^World Vision Ethiopia, Hawassa, Ethiopia; ^3^Department of Rural Development and Agricultural Extension, College of Agriculture, Hawassa University, Hawassa, Ethiopia

**Keywords:** adoption, quality protein maize, malnutrition, Misrak Badewacho, binary logit

## Abstract

This study aimed to identify the determinants of adoption of quality protein maize (QPM) varieties. QPM varieties are promoted as a solution to the problem of undernutrition, and their adoption is especially important in areas where maize is a nutritional staple food source. This study employed a cross-sectional design. A multistage sampling procedure was used to collect primary data from 143 sampled maize producers, which were analyzed using SPSS version 22. A binary logit model was used to identify major determinants. The results indicated that access to QPM seed, land size, on-farm income, involvement in off/non-farm activities, frequency of contact with development agents (Das), educational level of the household head, and participation of farmers on field days were statistically significant determinants of QPM variety adoption. Credit use was also a significant determinant of the adoption of QPM varieties but showed a negative influence. Therefore, this study recommends that all relevant stakeholders working at different levels in QPM production and extension pay attention to the factors that could affect farmers’ decisions to adopt QPM in the study area. Furthermore, all concerned bodies should work together to enhance the adoption of QPM varieties.

## 1 Introduction

Maize is a primary food crop that is grown in various agroecological zones. In sub-Saharan Africa (SSA), maize is consumed by people with different food preferences and socioeconomic backgrounds (1, 38). Maize was introduced to Ethiopia in the late 17th century and was mainly grown as subsistence crop (2). Recently, it has become a leading food source (3). Conventional maize varieties are highly produced in the country but are poor in protein quality due to being devoid of essential amino acids such as lysine and tryptophan (4, 5). The inability to obtain these essential amino acids from the daily diet results in acute malnutrition and may be a particular problem among young children, pregnant women, and lactating mothers whose diet is dominated by maize and who have limited alternative sources of these amino acids (6). Quality Protein Maize (QPM) has been developed to enhance lysine and tryptophan levels, potentially reducing deficiency risks by up to 21% (7, 8).

CIMMYT scientists have performed a series of maize breeding processes to develop better-quality maize since the mid-1960s from mutant maize genotypes that produce higher levels of lysine and tryptophan. These efforts have resulted in the development of quality protein maize (QPM) varieties (9). Malnutrition due to protein deficiency remains a problem in Ethiopia. Among children under five years, 44% are stunted in physical body growth, 22.6% are acute, and 29% are underweight (10, 39). In addition, 28% of child mortality is linked to undernutrition. Sixteen percent of all repetitions in primary schools are linked to stunting (40). The expenses related to malnutrition and diseases are high. The annual expected cost of undernutrition in Ethiopia has been $4.7 billion, which amounts to 16.5% of the gross domestic product (11).

Studies on the adoption of improved maize varieties among smallholder farmers in Ethiopia highlight several key factors. Ayele et al. (12) and Merga et al. (13) found that in central Oromia, adoption was positively influenced by education, household size, and access to credit, with adopters having larger family sizes and more land compared to non-adopters. Frequent contact with Development Agents (DAs) also positively impacted adoption decisions (12).

Beshir and Wegary (14) and Bekele (15) examined hybrid maize adoption in the drought-prone central rift valley and identified age of the household head, educational status, land size, and DA contact as influential factors, although DA contact was surprisingly negatively associated with hybrid maize adoption. These studies collectively indicate that education, household size, land size, access to credit, and interaction with DAs are crucial for the adoption of improved maize varieties (16). However, the impact of DA contact can vary depending on the context, emphasizing the need for targeted interventions and policies to enhance adoption rates among smallholder farmers in Ethiopia.

QPM varieties have been introduced and are promoted in the study area, in the Misrak Badewacho District of Southern Ethiopia, to reduce malnutrition in growing children, lactating mothers, and pregnant women. However, to the best of our knowledge, the acceptance and adoption of QPM varieties by farmers has not yet been studied or documented. Malnourishment in the study area could be due to the low level of adoption of the QPM varieties. Increasing the adoption of QPM varieties is possible if the influencing factors are addressed. Therefore, this study was conducted to identify the major determinants of QPM variety adoption in the Misrak-Badewacho District of Southern Ethiopia.

## 2 Methodology

### 2.1 Description of the study area

Misrak Badewacho District is located in the Hadiya zone of the South Nation and Nationality People Region (SNNPRS). The capital town, Shone, is located 337 km from Addis Ababa on the way towards Wolaita Sodo, passing through Halaba. It is also about 120 and 97 km from Hawassa and the zonal town of Hosaena, respectively. The astronomical location of Misrak Badewacho District is between 7°9ˈ00ˈˈ to 8°15ˈ00ˈˈ North latitude and 37° 5ˈ 00ˈˈ to 40° 00ˈ 00ˈˈ East longitude. The relative locations of the Misrak Badewacho District are Mierab Badewacho District to the west, Wolaita Zone to the south, Kembata-Tembaro Zone to the north, Halaba Zone to the northeast, and the Oromia regional State to the east. As shown in the Map below, Misrak Badewacho District does not share boundaries with other Districts of Hadiya Zone, except the Mierab Badewacho and Shone Town Administrations, because it is separated from other Districts of Hadiya Zone by the Kembata-Tembaro Zone.

The altitude of the Misrak Badewacho District ranges from 1,501 to 2040 m above sea level (*masl*). Agroecologically, it is in the range of dry and moist *woiyne dega*. The mean annual temperature ranges from 17.6 to 22.5 degree Celsius and its annual rainfall in millimeters (mm) ranges from 801 to 1,400. The total estimated population of the Misrak Badewacho District is 2′02’187, of which 1′00’226 (49.6%) are male and the remaining 1′01’961 (50.4%) are female. On the other hand, the number of rural households (HHs) is 2′9’427, and the population density per square kilometer (persons/km^2^) is 704 (17).

Agriculture is the major source of livelihood for the population in the study area. Maize is a leading food crop produced and consumed in the study area, followed by teff and haricot beans. The cultivation of maize spans 8,723 hectares, yielding an average of approximately 34.44 quintals per hectare (17). Food crops such as Irish potatoes, taro, and sorghum are also produced to some extent. Sugarcane, coffee, chills, and chats (*Catho edulis*) are the dominant cash crops in the study area.

### 2.2 Sample size and sampling procedure

Multistage sampling was used in this study. First, the Misrak Badewacho District was selected purposively, because of the popularity and potential of maize, availability of QPM varieties, prior knowledge of the researcher, and accessibility. In consultation with the Misrak Badewacho Woreda Agriculture and Natural Resource Development Office, three *kebeles* (the smallest administrative units after the district), namely Andegna Amburse, Amburse Anjulo, and Andegna Chafa, were selected out of 36 kebeles in the study area because of their potential as maize producers and the availability of QPM. Formulas by Cochran (18), Cochran et al. (19), Makr (20), Singh and Chaudhury (41), and Yamane (21) are popular statistical formulas that calculate sample sizes to determine an acceptable sample which can estimate results for the entire population with good precision. Among these, the Yamane formula was utilized in this study to minimize the availability of error and bias in determining the sample size for the survey. This is because Yamane’s formula is an approximation method of determining the sample size. A complete list of maize producers from the selected kebeles was then identified, and sample households were selected using a systematic random sampling procedure. Finally, because of time and resource limitations for the researcher, 143 sample respondents were selected for the interview schedule (Table 1).

**Table 1:** Sample kebeles and household size.

Sample kebele	Total maize producers	Sample households
Ambrose Anjulo	648	53
Andegna Amburse	502	42
Andegna Chafa	548	48
Total	1734	143

### 2.3 Source data and methods of data collection

**Figure 1:**
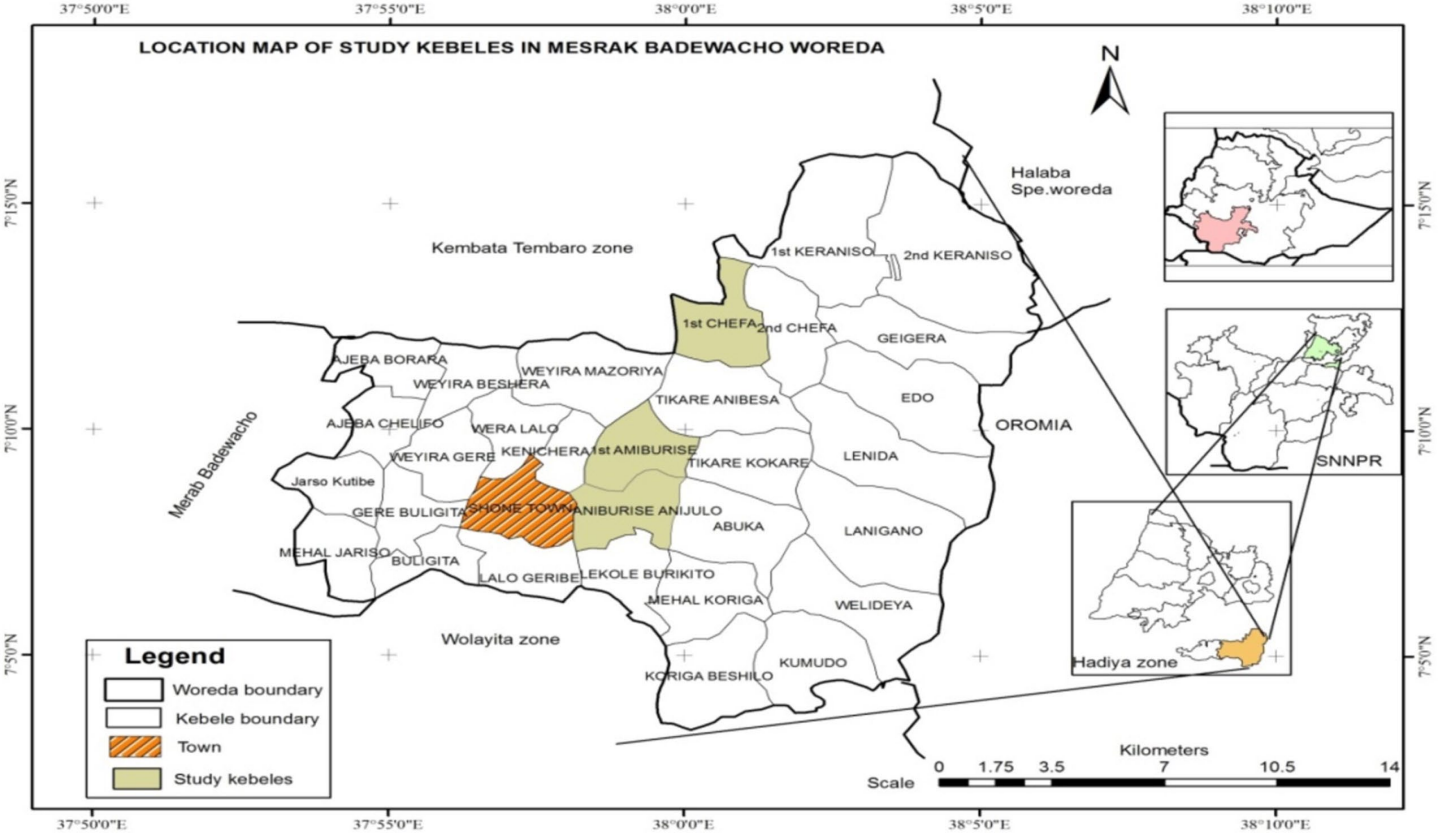
Map of study area. Source: (42).

This study used both primary and secondary data. Primary data were collected from the selected maize producers. Secondary data sources included published and unpublished information, research reports, scientific papers, journals, books, *Woreda* Agriculture and Natural Resource Development Office reports, and websites. Both quantitative and qualitative methods were used for data collection. Quantitative methods involved household surveys, while qualitative methods included key informant interviews (see Figure 1).

### 2.4 Methods of data analysis

Descriptive statistics such as means, standard deviations (SD), frequencies, and percentages were used to describe the socioeconomic features of the respondents. A binary logit model was employed to examine the determinants of adoption. It was used to predict the relative likelihood of QPM variety adoption because the dependent variable is dichotomous. The dependent variable “QPM variety adoption” has two possible outcomes—probabilities of a farmer to adopt and not to adopt QPM varieties; taking the value 1 or 0. A value of 1 indicates a farmer who adopted QPM varieties and 0 indicates a farmer who did not. Adopters of QPM varieties were farmers who planted one of the QPM varieties in the study area during the 2017 cropping season, while non-adopters were defined as farmers who did not plant QPM varieties. The model uses a cumulative logistic probability function, which better explains the underlying relationship between adoption decisions and the influencing factors. The advantage of this model is that the probabilities are bounded between zero and one, and it is simple to compute.

According to Gujarati (22), the logistic regression formula. and explain the Equations 1–7:


(1) $$P_{i}\;{\mathrm{= E(Y = 1 | X}}_{i})\;=\;\beta_{i}\;+\;\beta_{i}X_{i}$$

where:

Y = 1 means a given farmer adopts QPM varieties.

X_i_ is a vector of explanatory variables.

β_o_ is the constant and β_i_, i = 1, 2, 3…n are the coefficients of independent variables to be estimated.


(2) $$P_{i}=E\left(Y=1\;|\;X_{i}\right)=\frac{1}{e^{-\left(\beta 1+\beta 2\mathrm{Xi}\right)}}$$

This equation is rewritten as:-


(3) $$\frac{1}{1+e^{-zi}}\;=\;\frac{e^{z}}{1+e^{z}}$$

where Z_i_ = β_1_ + β_2_X_i_.

If (1-P_i_) is the probability of being a non-adopter, then P_i_ is the probability of adopting QPM varieties, and is given as follows:


(4) $$1-\mathrm{Pi}=\frac{1}{1+e^{-zi}}=e^{Zi}$$

Therefore, the equation can be rewritten as:


(5) $$\frac{\mathrm{Pi}}{1-\mathrm{Pi}}=\frac{1+e^{zi}}{1+e^{-zi}}=e^{zi}$$

Now, $\frac{\mathrm{Pi}}{1-\mathrm{Pi}}$ is simply the odds ratio in favor of adopting QPM. This is the ratio of the probability that a farmer will adopt QPM to the probability that a farmer will not adopt QPM varieties.

Now, if we take the natural log of Equation (5), the equation becomes:


(6) $$Li\;=\;\mathrm{In}\left(\frac{\mathrm{Pi}}{1-\mathrm{Pi}}\right)=Z_{i}=\beta_{1}+\beta_{2}X_{i}=\;\mathrm{In}\left(e^{\beta 0}+\overset{m}{\underset{i}{\sum }}\;\beta iXi\right)=Z_{i}$$

If the error term ε_i_ is taken into account the logit model becomes:


(7) $$\mathrm{Li = Zi =}\;\beta_{0}+\sum \mathrm{\beta iXi}+\varepsilon i$$

Accordingly, L_i_ is the log of the odds ratio, called the logit or logit model. Therefore, the logit model was employed to estimate the effect of the hypothesized independent variables on households’ decisions to use QPM varieties. Data were entered and analyzed using SPSS version 22.

According to Gujarati (22), multicollinearity occurs when the variance inflation factor (VIF) value is greater than 10 for continuous variables and the value of the contingency coefficient is greater than 0.75 for discrete variables. Accordingly, before the analysis and estimation of the model parameters, the existence of the problem of multicollinearity or association among continuous explanatory and discrete variables were checked using the VIF and contingency coefficient tests, respectively. No variables showed problems of multicollinearity (Tables 2, 3). Finally, as none of the variables showed multicollinearity problems, they were confidently included in the analysis model.

**Table 2:** Contingency coefficient for dummy and categorical variables.

Variables	Contingency coefficient
Sex of the household head	0.174
The educational level of the household head	0.473
The income level of the household	0.493
Involvement in off/non-farm activities	0.115
Participation in farmers’ field day	0.400
Participation in demonstrations	0.382
QPM seed access	0.441
Frequency of DA contact	0.408
Credit-Use	0.157
QPM marketability	0.133

**Table 3:** Variance inflation factor (VIF) for continuous variables.

Variable	VIF	1/VIF
Age at last birthday	1.03	0.970903
Household size in number	1.05	0.949456
Land size in Timad	1.08	0.925521
Mean VIF	1.05	

*Timad: this is the type of measurement locally used by farmers to define land size.

### 2.5 Dependent and independent variables

The dependent variable for the logistic model was a dummy variable indicating whether the household head adopted QPM varieties. In this study, the dependent variable was the adoption of QPM varieties, with a value of 1 or 0. Adopters of QPM varieties were defined in this study as farmers who planted at least one of the QPM varieties in the study area in the 2017 cropping season, and non-adopters were farmers who did not grow QPM varieties in the 2017 cropping season or those who discontinued it. The independent or explanatory variables explain and influence the dependent variable. Based on various studies, the adoption of QPM varieties is influenced by sociodemographic, economic, and institutional factors, which are explained in detail in Table 4.

**Table 4:** Summary of independent variables, measurement, and hypothesized sign.

Variable code	Type	Description	Unit and expected sign
Age	Continuous	Age of household head	At last birthday (−)
Educ	Categorical	Educational level of the household head	No formal 0, primary 1, secondary, 2 and above secondary 3 (+)
HH size	Continuous	Household Size	Household size in number (+)
Farming	Categorical	On-farm Income in Birr	1 = Less than 10,000, 2 = 10,000-20,000, 3 = 20,000-30,000, 4 = 30,000–40,000 and 5 = above 40,000 Birr (+)
L size	Continuous	Land size	Timid (+)
Of arm	Dummy	Involvement in off /nonfarm activities	If yes 1 otherwise 0 (+)
Field day	Dummy	Attended QPM field days	If yes 1 otherwise 0 (+)
Demo	Dummy	Attended QPM demonstrations	If yes 1 otherwise 0 (+)
Seed	Dummy	Availability of QPM seed on time	If yes 1 otherwise 0 (+)
DAcont	Dummy	Frequency of DA contact	If no contact =0, 1 = once, 2 = twice, 3 = three time and 4 = more than 3 times per month (+)
Credit	Dummy	Credit use	If yes 1 otherwise 0 (+)
Marketable	Dummy	Marketability of QPM	If yes 1 otherwise 0 (+)

## 3 Results and discussion

### 3.1 Descriptive statistical analysis results

#### 3.1.1 Awareness and extent of QPM variety adoption

The decision to adopt innovation begins when an individual is aware of a certain technology and has the necessary information before adopting it. The results in Table 5 indicate that the degree of awareness of QPM varieties among respondents was high. From a total of 143 respondents, 106 (74.1%) comprising both adopter and non-adopter respondents were aware of QPM varieties. This could be the result of QPM farmers’ field days, demonstrations, local dishes, and the frequency of contact with development agents (Das).

**Table 5:** Level of awareness extent of QPM varieties adoption.

Aware about QPM	Adopters		Non-adopters		Total	
	Frequency	Percent	Frequency	Percent	Frequency	Percent
Yes	38	100	68	64.8	106	74.1
No	0	0	37	35.2	37	25.9
Total	38	100	105	100	143	100

The extent of adoption was measured as the percentage of the sample households that grew QPM varieties during the 2017 cropping calendar. QPM was introduced in the study area a decade ago. Among the sampled households (143), only 38 of them (26.6%) adopted QPM varieties in the study area. The remaining 73.4% did not adopt QPM varieties during the above mentioned cropping season (Table 5). This result reveals that the level of QPM variety adoption was too low in the study area. The major reasons for the low adoption of QPM varieties were the seed access problem, the low yield potential of QPM compared with conventional maize varieties, the problem of a better market for QPM, and the lower resistance of QPM to diseases (see Table 6).

**Table 6:** Reasons for not adopting QPM varieties.

Reasons for non-adopting	Frequency	Percent
QPM seed access problem	37	35.2
Lack of knowledge	27	25.7
QPM is less productive	15	14.3
QPM is less marketable	12	11.4
QPM is less resistant to disease	9	8.6
Others	5	4.8
Total	105	100

### 3.2 Determinants of adoption of QPM varieties

#### 3.2.1 QPM seed access

Availability of QPM seeds on time at an affordable price at the community level plays a significant role in technology adoption. Table 7 shows that access to seeds was statistically significant at less than 1% level and was positively related to QPM variety adoption. In addition, the results of the odds ratio revealed that if households’ access to QPM seed varieties is favorable, the probability of adoption of QPM varieties increases by 63.797. This result is consistent with those reported by Yishak and Punjabi (23) and Gregory (24).

**Table 7:** The maximum likelihood estimation of the binary logit model (*N* = 143).

Variable	B	S.E.	Wald	Df	Sig.	Exp(B)
Sex	0.042	1.572	0.001	1	0.979	1.043
Age	−0.219	0.166	1.756	1	0.185	0.803
Educ	1.596	0.871	3.354	1	0.067^*^	4.933
HHsize	0.413	0.413	0.999	1	0.318	1.511
Farming	1.570	0.653	5.779	1	0.016^**^	4.807
Lsize	1.652	0.630	6.874	1	0.009^***^	5.216
Offfarm	3.307	1.382	5.730	1	0.017^**^	27.305
Fieldday	2.988	1.684	3.147	1	0.076^*^	19.851
Demo	1.551	1.413	1.205	1	0.272	4.716
Seed	4.156	1.528	7.397	1	0.007^***^	63.797
DAcont	1.998	0.910	4.820	1	0.028^**^	7.375
Credit	−2.765	1.618	2.918	1	0.088^*^	0.063
Marketable	−0.878	1.473	0.356	1	0.551	0.415
Constant	−18.476	6.949	7.070	1	0.008	0.000

−2 Log likelihood = 28.116. LR chi^2^ = 137.47. Cox and Snell R Square = 0.618. Nagelkerke R Square = 0.900. Prob > chi2 = 0.0000. Correctly predicted = 96.5%. ***, **, and * indicate significant at the 1, 5, and 10% levels, respectively.

#### 3.2.2 Land size

It was found that land size positively and significantly influenced the probability of adoption of QPM varieties at less than 1% significance level. This is because farmers with large land areas have available land to expand the areas for the introduced varieties. This result implies that farmers with larger land areas are more likely to adopt QPM varieties than farmers with smaller land areas. The odds ratio of 5.216 for land size shows that, other things being constant, the odds ratio in favor of adopting QPM varieties increased by a factor of 5.216 as farm size increased over time (Table 7). This result is consistent with those reported by Beshir and Wegary (14), Wangare (25), and Thomson et al. (26).

#### 3.2.3 On-farm income

Income of the Household from on-farm sources was statistically significant at less than 5% level and positively linked to the adoption of QPM varieties. The odds ratio of 4.807 in this respect revealed that the other variables remained the same; the odds in favor of adopting QPM varieties increased by a factor of 4.807 as on-farm income increased by one birr (Table 7). These findings are similar to those reported by Felistus (27) and Raphael (28).

#### 3.2.4 Involvement in off/non-farm activities

Households’ involvement in off/non-farm activities helps them earn income and purchase inputs. Therefore, as expected, the involvement of the households in off/non-farm activities had a positive influence on the adoption of QPM varieties at less than 5% significance level. Hence, the odds ratio of 27.305 for involvement in off/non-farm activities indicates that, other things being constant, the odds ratio in favor of adopting QPM varieties increased by a factor of 27.305 for households involved in off/non-farm activities (Table 7). These findings are consistent with those of Katengeza et al. (29).

#### 3.2.5 Frequency of contact with DAs

Frequency of contact with DAs is important for sustainably acquiring new skills and knowledge of technologies. Therefore, the frequency of contact with DAs positively influenced the adoption of QPM varieties at less than 5% significance level. Hence, the results of the logit model revealed that the odds ratio in favor of households’ adoption of QPM varieties was 7.375. This is because the frequency of contact with DAs increases the probability of obtaining updated information on new agricultural technologies. Thus, farmers with frequent contact with DAs are more likely to adopt new technologies than those with less contact with DAs. These findings are in line with those of Bamire and Adebayo (30). However, the results of Beshir et al. (31) and Ademiluyi (32) contradict this finding, as the frequency of DA contact was negatively associated with technology adoption.

#### 3.2.6 Educational level of the household head

Educated farmers are more capable of processing information and assessing the relative advantages of new technologies. The logit model results indicate that the educational level of the household head was positively and significantly associated with the probability of adoption of QPM varieties at less than 10% significance level. The odds ratio of 4.933 for educational level indicates that as the educational level increases by one level, the odds ratio in favor of adopting QPM varieties increases by a factor of 4.933, with other factors remaining the same. This finding is consistent with those of Salifu and Salifu (33), Ebojei et al. (34), Abadi (35), and Hussein and Abukari (36), who found that education had a positive relationship in their studies.

#### 3.2.7 Attending farmers’ field days

It was found that exposure to information due to attending field days had positively and significantly influenced the probability of adoption of QPM varieties at the 10% significance level. Hence, the result of the odds ratio in Table 7 explains that, other factors remaining constant, households’ participation in farmers’ field days increases the probability of adoption of QPM varieties by 19.851. This was because farmers attending field days for QPM gained better knowledge, which contributed to their adoption of QPM varieties in the study area. This result is consistent with the findings of Gregory and Sewando (9).

#### 3.2.8 Credit use

Credit use is assumed to positively and significantly influence the adoption of the QPM variety. In this study, credit use was significant at less than 5% level but negatively related to the adoption of QPM varieties (Table 7). This result was unexpected and contrary to the economic theory. Consequently, the odds ratio result shows that households’ credit use decreases the probability of adopting QPM varieties by 0.063. This is because credit was not invested in purchasing the QPM seeds or fertilizers. This result disagrees with that of Abadi et al. (43) and Damas and Moti (37).

## 4 Conclusion and policy implications

Malnutrition problems still exist in the study area and can be reduced by increasing the adoption of QPM varieties. An increase in the adoption of QPM varieties is possible if the factors affecting adoption are addressed. This study presents the results from a descriptive analysis and binary logit model to identify the determinants of the adoption of QPM varieties. According to the results of descriptive statistics, the major constraints for the adopting QPM varieties were the QPM seed access problem, low productivity of QPM compared with conventional maize, similar price for QPM and conventional maize, lack of better markets for QPM, and low resistance of QPM to diseases.

The results of the binary logistic regression model indicated that access to QPM seed, land size, income from on-farm sources, involvement of households in off/non-farm activities, frequency of DA contact, educational level of the household head, and farmers’ field days significantly and positively determined the adoption of QPM varieties. However, credit use negatively affected the adoption of QPM, as credit was not used to purchase QPM seeds and fertilizers.

Therefore, this study recommends that the Bureau of Agriculture at different levels, researchers, QPM seed producers, policymakers, non-government development actors, policymakers, and other relevant stakeholders working at different levels in QPM production and extension should pay attention to the factors that could affect farmers’ decisions to adopt QPM in the study area. It is also recommended that researchers in the field of plant breeding should be improving the characteristics of QPM, particularly in terms of achieving high productivity and resistance to pests and diseases.

Furthermore, all concerned bodies should work together to enhance the adoption of QPM varieties.

## Data Availability

The original contributions presented in the study are included in the article/supplementary material, further inquiries can be directed to the corresponding author.
